# Measurement of the Verdet Constant of Polarization-Maintaining Air-Core Photonic Bandgap Fiber

**DOI:** 10.3390/s17081899

**Published:** 2017-08-17

**Authors:** Ningfang Song, Xiaoyang Wang, Xiaobin Xu, Wei Cai, Chunxiao Wu

**Affiliations:** Department of Opto-electronics Engineering, Beihang University, Beijing 100191, China; Songnf@buaa.edu.cn (N.S.); xiaoyang_wang@buaa.edu.cn (X.W.); sdfz174caiwei@126.com (W.C.); wuchunxiao34@126.com (C.W.)

**Keywords:** Faraday effect, gyroscopes, microstructured fibers

## Abstract

We propose a method based on the white-light interference technique for measuring the Verdet constant of a polarization-maintaining air-core photonic bandgap fiber (PM-PBF). The experimental results show that the Verdet constant of the PM-PBF is ~3.3 mrad/T/m for the broadband light with a spectral width of ~38 nm and a mean wavelength of ~1550 nm, which is ~124 times less than that of a conventional stress-induced birefringent fibers called PANDA fibers (~0.41 rad/T/m for the same broad-spectrum light). The results indicate that the nonreciprocal error induced by the Faraday effect in a fiber optic gyroscope (FOG) made of the PM-PBF is theoretically ~25 times less than that of a conventional FOG made of the PANDA fiber when other conditions, such as the fiber twist, fiber coil area, and so on, are the same.

## 1. Introduction

The Faraday effect may deteriorate the performance of a fiber optic gyroscope (FOG) when the sensing coil of the FOG is exposed to a geomagnetic field [1,2]. Protecting the coil with a magnetic shield is the strategy usually adopted to reduce the impact of the Faraday effect on the FOG performance. The magnetic shield, however, introduces additional weight, cost, and complexity, and, as a result, limits the FOG’s applications. 

Photonic crystal fiber (PCF) is a class of optical fiber based on two-dimensional photonic crystal, and strongly attracts researchers’ interest owing to its special characteristics. New PCFs emerge constantly according to different applications, such as the recent composite PCFs [3,4], nodeless air-core PCFs [5], and so on. Among those fibers, air-core photonic bandgap fiber (PBF) offers a radically new method for solving the problem of environment (including magnetic field) adaptation, because the PBF causes light to propagate in air that exhibits much lower sensitivity to magnetic fields than conventional SiO_2_ [6]. Thus, a polarization-maintaining air-core PBF (PM-PBF) is expected to be more effective in reducing the Faraday effect in a FOG [7].

A. M. Smith [8,9] measured the Verdet constant of a single-mode optical fiber, and found it to be ~3.61 rad/T/m at 632.8 nm and ~2.05 rad/T/m at 830 nm. J. L. Cruz et al. [10] measured the effective Verdet constant of a standard single-mode fiber and found it to be 0.54 ± 0.02 rad/T/m at 1523 nm. J. Noda et al. [11] measured the Verdet constant of a conventional PANDA fiber at different wavelengths, showing it to be ~0.60 rad/T/m at 1550 nm. H. Wen et al. [12] reported the first measurement of the Verdet constant of a seven-cell non-PM PBF, 6.1 ± 0.3 mrad/T/m at 1545 nm, which is about 90 times less than that of standard single-mode fiber (Corning’s SMF-28). L. Sun et al. [13,14] measured the effective Verdet constant of a 25 wt. % terbium-doped-core phosphate fiber and a 56 wt. % terbium (Tb)-doped silicate fiber, which were found to be −6.2 ± 0.4 rad/T/m and −24.5 ± 1.0 rad/T/m at 1053 nm, respectively. Although the Verdet constants of many kinds of fibers have been reported, the Verdet constant of a PM-PBF remains unreported. What is more, the light source used in previous studies is a laser with a narrow spectral width, and the obtained Verdet constant is only credible at a certain wavelength, which is not suitable for an accurate analysis of the nonreciprocal error induced by the Faraday effect in a FOG, because there is always a broadband light source with a spectral width of tens of nanometers employed in a FOG.

Therefore, in this paper, we report the measurement of the Verdet constant of a PM-PBF using the white-light interference technique based on a broad-spectrum amplified spontaneous emission (ASE) source and an all-fiber setup. This result can provide a foundation for the analysis of both the magnetic properties in a PM-PBF and nonreciprocal error induced by the Faraday effect in a polarization-maintaining air-core photonic-bandgap FOG (PM-PBFOG).

## 2. Measurement Principle

**Figure 1:**
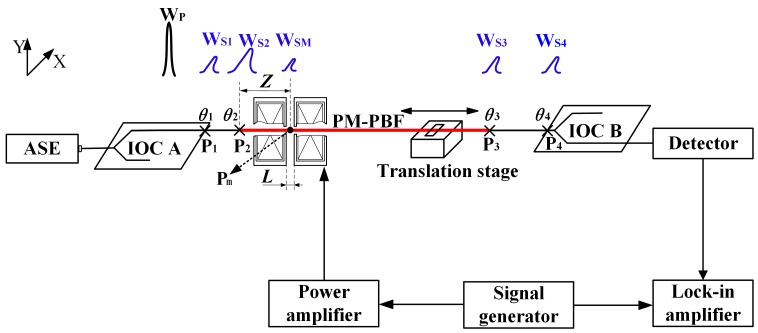
Schematic of the setup for measuring the Verdet constant of the polarization-maintaining air-core photonic bandgap fiber (PM-PBF). The bold red line between P_2_ and P_3_ is the PM-PBF.

The schematic of the Verdet constant measuring setup is shown in Figure 1, which is based on the configuration of Reference [11]. Light from an ASE source is launched into integrated optic chip (IOC) A. The IOC has an extinction ratio of more than 70 dB, so the output light becomes a linear polarization beam W_p_. Next, W_p_ is coupled into the PM-PBF (~60 cm) under test. The PM-PBF passes through a ~1 mm-diameter bore in the center of an electromagnet, and the width of the pole-gap of the electromagnet is L ≈ 1 mm. The electromagnet is placed near the fusion splicing point P_2_ and excited by a sinusoidal electrical signal, generated by a power amplifier connected to a signal generator. Meanwhile, the PM-PBF is fixed on a one-dimensional motorized translation stage to realize precise movements in the magnetic field. Finally, the light is converted to an electronic signal by the detector after passing through IOC B. A lock-in amplifier is used to demodulate this electronic signal and resolve the Verdet constant of the PM-PBF.

In Figure 1, P_1_, P_2_, P_3_, and P_4_ are the coupling or fusion splicing points between IOC A and its pigtail, IOC A’s pigtail and the PM-PBF, the PM-PBF and IOC B’s pigtail, and IOC B’s pigtail and IOC B, respectively. The alignment angles between birefringent axes of the related two parts at the points P_1_, P_2_, P_3_, and P_4_ are assumed to be *θ*_1_, *θ*_2_, *θ*_3_, and *θ*_4_, respectively. *θ*_1_, *θ*_2_, and *θ*_4_ are around 0°; *θ*_3_ is about 90°. When the polarized primary wave W_P_ propagates through P_1_, P_2_, P_3_, and P_4_, the polarization crossover-induced secondary waves W_S1_, W_S2_, W_S3_, and W_S4_ are produced, with a polarization state perpendicular to that of W_P_, because the cross-coupling exists at those coupling or fusion splicing points. Meanwhile, an equivalent rotation angle *θ*_m_ is caused by the Faraday effect at the point P_m_ within the electromagnet, so another secondary wave W_SM_ also arises when W_p_ passes through the point P_m_ [11]. Considering the polarization states of the primary and secondary waves, we find that the primary wave W_P_ is eliminated by the polarizer within IOC B, and only W_S1_, W_S2_, W_S3_, W_S4_, and W_SM_ are able to arrive at the detector; their optical paths are shown in Table 1. The amplitude of W_SM_ reflects the Verdet constant of the PM-PBF, so the purpose of this work is to resolve the amplitude of W_SM_.

n_s1_ and n_f1_, n_s2_ and n_f2_, n_s3_ and n_f3_ denote the refractive index of the slow and fast axes of IOC A’s pigtail, PM-PBF, and IOC B’s pigtail, respectively. L_P1-P2_, Z, L_Pm-P3_, and L_P3-P4_ denote the distance between P_1_ and P_2_, P_2_ and P_m_, P_m_ and P_3_, as well as P_3_ and P_4_, respectively.

If the source is the laser that was used in References [8,9,10,11,12,13,14], all of these wavetrains (W_S1_, W_S2_, W_S3_, W_S4_, and W_SM_) at the detector would interfere and the situation would be very complicated. However, as an ASE source with a very short coherence length (~61.9 μm) is used here, we can make the optical path difference sufficiently small between W_SM_ and W_S2_ (see Table 1), which means that the interference only occurs between W_SM_ and W_S2_, by optimizing the location of the electromagnet and lengths of the PM-PBF, IOC A’s pigtail, and IOC B’s pigtail. As a result, W_S2_ actually becomes a reference wavetrain to interfere with the signal wavetrain W_SM_. Then, the situation becomes very simple, and the interference intensity between W_S2_ and W_SM_ at the detector is given by:

(1) $$V_{\mathrm{interference}}=V_{S2}+V_{\mathrm{SM}}+2\sqrt{V_{S2}V_{\mathrm{SM}}}\gamma \left(\frac{\Delta \phi \lambda }{2\pi c}\right)\cos\Delta \phi$$

where *V*_S2_ and *V*_SM_ are voltages corresponding to W_S2_ and W_SM_ at the detector, respectively, and they are given by the first two formulas in Equation (2). In addition, *γ* is the coherence function of the ASE source [7], Δ*ϕ* is the phase difference between W_S2_ and W_SM_ (given by the third formula in Equation (2)), *λ* is the mean wavelength of the source, and *c* is the velocity of light.

(2) $$\{\begin{array}{l} V_{S2}=\left(P_{0}R/\alpha_{1}\alpha_{2}\right)\cos^{2}\theta_{1}\cdot \sin^{2}\theta_{2}\\ V_{\mathrm{SM}}=\left(P_{0}R/\alpha_{1}\alpha_{2}\right)\cos^{2}\theta_{1}\cdot \cos^{2}\theta_{2}\cdot \sin^{2}\theta_{m}\\ \Delta \phi =2\pi Z/L_{B} \end{array}$$

In Equation (2), *P*_0_ is power of the ASE source, *R* is the conversion efficiency of the detector, α_1_ denotes the loss from the ASE source to the point P_m_, α_2_ denotes the loss from the point P_m_ to the detector, and *Z* is the distance between the points P_2_ and P_m_. *θ*_m_ caused by the Faraday effect is *VB*sin (*ωt*) *L*sin (*δ*/2)/(*δ*/2) [11], where *V* is Verdet constant of the fiber, *B* is intensity of the magnetic field and is approximately constant within the pole gap, *ω* is modulation frequency of the magnetic field applied to the PM-PBF, *δ* = 2π*L*/*L*_B_, and *L*_B_ is beat length of the fiber.

The extremely low magnetic-field sensitivity of the PM-PBF causes *θ*_m_ and *V*_SM_ to be extremely small, so it is impossible for the direct detection of the interference signal *V*_interference_. Thus, coherence detection must be applied here. A sinusoidal magnetic field is applied to the PM-PBF at the point P_m_ to modulate *V*_SM_ and the interference signal (*V*_interference_), and only the third term of Equation (1) can be resolved because only this term has the same frequency component with the modulation frequency *ω* [15]. Then, the demodulation signal (*V*_demodulation_) resolved by the lock-in amplifier is given by:

(3) $$V_{\mathrm{demodulation}}\approx \frac{P_{0}RVBL_{B}\gamma \left(\frac{Z\lambda }{L_{B}c}\right)\sin\left(\frac{\pi L}{L_{B}}\right)\cdot \cos^{2}\theta_{1}\cdot \sin2\theta_{2}\cdot \cos\left(\frac{2\pi Z}{L_{B}}\right)}{\sqrt{2}\pi \alpha_{1}\alpha_{2}}$$

Thus, *V*_demodulation_ demonstrates an approximately sinusoidal oscillation as *Z* changes linearly. Furthermore, its maximum peak-to-peak value (*V*_demodulation-p-to-p_) reveals the Verdet constant (*V*) of the PM-PBF to be:

(4) $$V\approx \frac{\pi \alpha_{1}\alpha_{2}V_{\mathrm{demodulation}-p-\mathrm{to}-p}}{\sqrt{2}P_{0}RBL_{B}\sin\left(\frac{\pi L}{L_{B}}\right)\cdot \cos^{2}\theta_{1}\cdot \sin2\theta_{2}}$$

Compared with the other methods in previous studies used to determine the Verdet constant [8,9,10,11,12,13,14], not a laser source but a broad-spectrum ASE source and the white-light interference technique is used here, so the measured Verdet constant is more accurate and can be applied to the analysis of the nonreciprocal error induced by the Faraday effect in a FOG. Meanwhile, the ASE source has a very short coherence length, so it can avoid unwanted interference between irrelevant secondary waves, which is very important to simplify the analysis and improve the measurement system performance. Moreover, all optical components of the measuring setup employ all-fiber connections, which can avoid secondary waves induced by interface reflection and improve the system reliability and stability.

## 3. Experimental Results

**Figure 2:**
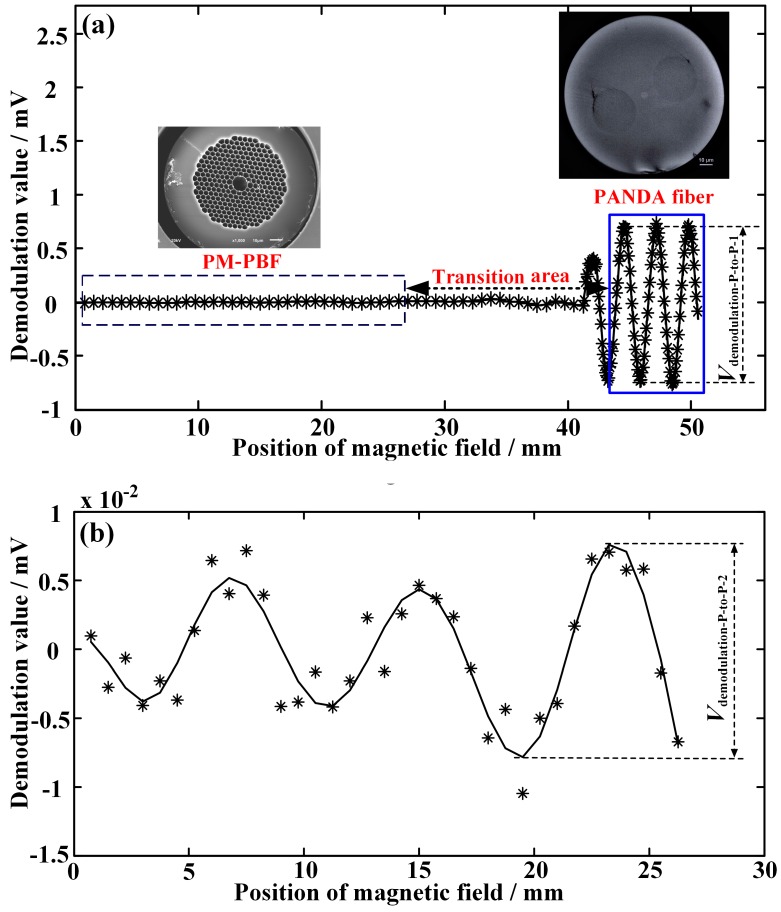
Test results of the Verdet constants of the PM-PBF and conventional PANDA fiber. (**a**) *V*_demodulation_ sinusoidally oscillates as the position (P_m_) of the magnetic field uniformly scans from the right to the left of the fusion splicing point P_2_ in Figure 1. (**b**) Enlarged image of the area within the dashed-line rectangle in (**a**).

An experiment setup based on Figure 1 is established to measure the Verdet constant of a commercial seven-cell PM-PBF (see the left inset in Figure 2a) with an air filling ratio of ~97%, a loss of ~25 dB/km, a mode diameter of ~9 um, and a cladding diameter of ~120 um, respectively. The ASE source has a flat spectrum with a width of ~38 nm and a mean wavelength of ~1550 nm. The detector’s conversion efficiency R is ~0.038 V/μW. The fusion splicing angle *θ*_1_ ≈ 1°, and *θ*_2_ is deliberately made larger (~6°) to increase the intensity of the reference wavetrain W_S2_ but avoid saturation of the detector at the same time. The magnetic-field intensity *B* is modulated by a 30-Hz sinusoidal signal, and has an amplitude of ~0.25 T as a result.

When the PM-PBF is precisely moved forward by the translation stage to make the magnetic position (P_m_) uniformly scan from the right to the left of the fusion splicing point P_2_ (see Figure 1), the demodulation value (*V*_demodulation_) of the interference signal (*V*_interference_) is presented in Figure 2a. The demodulation value within the dashed- and solid-line rectangle in Figure 2a corresponds to the PM-PBF (see the left inset) and conventional PANDA fiber (see the right inset) that has a loss of ~0.27 dB/km, a mode diameter of ~6.5 um, and a cladding diameter of ~125 um, respectively. Furthermore, it is interesting to note that there is a transition area in the middle which seems to be a little disorganized. This is reasonable because the states of the fibers, especially the air holes in the PM-PBF, have changed in the process of fusion splicing. Therefore, the experimental results within this area are not accurate for the analysis of the Verdet constant of the PM-PBF and conventional PANDA fibers, and we utilize the data slightly farther from this area. For the conventional PANDA fiber, as illustrated in the solid-line rectangle in Figure 2a, *V*_demodulation_ sinusoidally varies with the position P_m_ of the magnetic field imposed on the fiber, which agrees well with the theoretical expectation in the previous section. It shows that the oscillation period is ~2.6 mm, which actually means that the beat length *L*_B_ equals ~2.6 mm [16]. The peak-to-peak value of the demodulation signal is *V*_demodulation-P-to-P-1_ = ~1.42 mV. Therefore, the Verdet constant of the conventional PANDA fiber is ~0.41 rad/T/m based on Equation (4).

For the PM-PBF, the enlarged image of the corresponding demodulated signal within the dashed-line rectangle in Figure 2a is shown in Figure 2b. Obviously, the oscillation period is ~7.75 mm, which means that the beat length of the PM-PBF is ~7.75 mm [16]. The peak-to-peak value of the demodulation signal is *V*_demodulation-P-to-P-2_ = ~17.5 μV. Consequently, the Verdet constant of the PM-PBF equals ~3.3 mrad/T/m, which is ~124 times less than that of the conventional PANDA fiber.

The experiment is performed at room temperature, and the most sensitive component to temperature is the birefringence of the polarization-maintaining fiber. However, the temperature-variation-induced error of the Verdet constant can be ignored because it is a few orders of magnitude smaller than the absolute value. According to the theoretical analysis in Reference [2], the nonreciprocal error induced by the Faraday effect in a FOG is proportional to the ratio of the Verdet constant (*V*) to the birefringence (Δ*β*). Therefore, based on our measurement results of the Verdet constant and beat length, the nonreciprocal error induced by the Faraday effect in a PM-PBFOG is theoretically ~25 times less than that of a conventional FOG made of PANDA fiber coil when the other conditions, such as the fiber twist, fiber coil area, and so on, are the same. Similarly, this error in PM-PBFOG is theoretically ~19 times less than that of a FOG made of a seven-cell non-PM PBF [12].

## 4. Conclusions

In summary, we proposed a method based on the white-light interference technique to measure the Verdet constant of a PM-PBF. The setup employs an ASE source with a spectral width of ~38 nm and a mean wavelength of ~1550 nm. The results show that the Verdet constants of the PM-PBF and the conventional PANDA fiber are, respectively, ~3.3 mrad/T/m and ~0.41 rad/T/m, indicating that the nonreciprocal error induced by the Faraday effect in a FOG made of the PM-PBF is theoretically ~25 times less than that of a conventional FOG made of the PANDA fiber when the other conditions, such as the fiber twist, fiber coil area, and so on, are the same. Therefore, the experimental results provide an important foundation for the analysis of the magnetic properties of a PM-PBF and the Faraday effect in a PM-PBFOG.

## Figures and Tables

**Table 1:** Optical path of secondary waves.

Secondary Waves	Optical Path
W_S1_	n_s1_L_P1-P2_ + n_s2_(Z + L_Pm-P3_) + n_f3_L_P3-P4_
W_S2_	n_f1_L_P1-P2_ + n_s2_(Z + L_Pm-P3_) + n_f3_L_P3-P4_
W_SM_	n_f1_L_P1-P2_ + n_f2_Z + n_s2_L_Pm-P3_ + n_f3_L_P3-P4_
W_S3_	n_f1_L_P1-P2_ + n_f2_(Z + L_Pm-P3_) + n_f3_L_P3-P4_
W_S4_	n_f1_L_P1-P2_ + n_f2_(Z + L_Pm-P3_) + n_s3_L_P3-P4_
